# A Distinct and Divergent Lineage of Genomic Island-Associated Type IV Secretion Systems in *Legionella*

**DOI:** 10.1371/journal.pone.0082221

**Published:** 2013-12-16

**Authors:** Bryan A. Wee, Megan Woolfit, Scott A. Beatson, Nicola K. Petty

**Affiliations:** 1 Australian Infectious Diseases Research Centre and School of Chemistry and Molecular Biosciences, University of Queensland, Brisbane, Queensland, Australia; 2 School of Biological Sciences, Monash University, Melbourne, Victoria, Australia; 3 The ithree institute, University of Technology Sydney, Ultimo, New South Wales, Australia; INRA Clermont-Ferrand Research Center, France

## Abstract

*Legionella* encodes multiple classes of Type IV Secretion Systems (T4SSs), including the Dot/Icm protein secretion system that is essential for intracellular multiplication in amoebal and human hosts. Other T4SSs not essential for virulence are thought to facilitate the acquisition of niche-specific adaptation genes including the numerous effector genes that are a hallmark of this genus. Previously, we identified two novel gene clusters in the draft genome of *Legionella pneumophila* strain 130b that encode homologues of a subtype of T4SS, the genomic island-associated T4SS (GI-T4SS), usually associated with integrative and conjugative elements (ICE). In this study, we performed genomic analyses of 14 homologous GI-T4SS clusters found in eight publicly available *Legionella* genomes and show that this cluster is unusually well conserved in a region of high plasticity. Phylogenetic analyses show that *Legionella* GI-T4SSs are substantially divergent from other members of this subtype of T4SS and represent a novel clade of GI-T4SSs only found in this genus. The GI-T4SS was found to be under purifying selection, suggesting it is functional and may play an important role in the evolution and adaptation of *Legionella*. Like other GI-T4SSs, the *Legionella* clusters are also associated with ICEs, but lack the typical integration and replication modules of related ICEs. The absence of complete replication and DNA pre-processing modules, together with the presence of *Legionella*-specific regulatory elements, suggest the *Legionella* GI-T4SS-associated ICE is unique and may employ novel mechanisms of regulation, maintenance and excision. The *Legionella* GI-T4SS cluster was found to be associated with several cargo genes, including numerous antibiotic resistance and virulence factors, which may confer a fitness benefit to the organism. The *in-silico* characterisation of this new T4SS furthers our understanding of the diversity of secretion systems involved in the frequent horizontal gene transfers that allow *Legionella* to adapt to and exploit diverse environmental niches.

## Introduction

Type IV secretion systems (T4SSs) are a highly diverse family of complex macromolecular structures involved in a variety of functions, including conjugation, protein translocation and DNA uptake and release (for a review, see [1]). Attempts to classify T4SSs into subtypes have been based on function and the type of substrate (DNA or protein) translocated, or phylogenetic relationships between shared representative genes [1], [2]. However, these classification schemes are hampered by the diversity of functions and structures of the T4SS family as well as the mosaic nature of the genomic loci that encode them, which have evolved though extensive recombination and modular exchange. Nevertheless, the different subtypes of T4SSs share common mechanisms, alongside specialised properties unique to certain subtypes or bacteria [1].

Based mainly on sequence similarity and gene organisation, T4SSs in Proteobacteria have been categorized into three main types: type IVA, type IVB, and genomic island-associated (GI) T4SSs. Members of the type IVA (T4ASS) are related to the archetypal *Agrobacterium tumefaciens* VirB/D4 system, and further divided into subtypes F and P according to their similarity to representative plasmid conjugation systems of the incompatibility groups IncF and IncP [2]–[4]. Type IVB secretion systems (T4BSS), also known as type I based on similarity to conjugative IncI plasmids, are divergent from the T4ASSs and related to the archetypal Dot/Icm (Defective in organelle trafficking/Intracellular multiplication) protein translocation system of *Legionella pneumophila* and *Coxiella burnetti* [3], [5]. A third distinct class of T4SSs was found to be associated exclusively with genomic islands and called GI type or genomic island-associated T4SS (GI-T4SS) [6].

GI-T4SSs were initially identified as associated with a group of related genomic islands including the pKLC102 and PAPI elements in *Pseudomonas aeruginosa*, the *clc* element in *Pseudomonas knackmussii* B13, the ICE*Hin*1056 element in *Haemophilus influenzae* and the SPI-7 island of *Salmonella enterica* serovar Typhi [6]–[10]. These islands have all now been classified as integrative and conjugative elements (ICEs), a family of self-transmissible genomic elements [11], [12]. Usually found integrated at specific sites in the host chromosome, ICEs are a class of mobile genetic element capable of self-encoded excision, circularization, replication, conjugal transfer and integration into recipient bacterial chromosomes. ICEs share a common genomic structure, with conserved core modules of genes encoding functions for integration, replication and transfer, interspersed with varying types and numbers of cargo genes, which are not required for the function of the ICE [11]–[13]. Many of these elements carry cargo genes that confer a fitness advantage to the host, for example SPI-7 encodes a major virulence factor of *S.* Typhi, the Vi exopolysaccharide capsule [14], ICE*Hin*1056 carries multiple antibiotic resistance genes [7], and different ICEs have been shown to be directly responsible for the highly virulent phenotypes of several *P. aeruginosa* strains [15]–[17].

GI-T4SSs are located in the transfer modules of ICEs and encode the conjugation machinery required for transfer of the associated element. Knockout studies have shown that many of the genes encoded in the GI-T4SS are essential for conjugation of ICEs, while others greatly enhance the frequency of ICE transfer via conjugation to recipient cells [6], [11], [18]. Additionally, electron microscopy studies have shown that the GI-T4SS is responsible for the production of a single long, thin pilus per cell in *H. influenzae* [6], [19].

The Gram-negative pathogen *Legionella pneumophila* encodes several different T4SSs, which are thought to have contributed to the large number of horizontally acquired non-essential genes found in the genome of this species [20], [21]. While the effector-translocating Dot/Icm T4BSS is known to be essential for the parasitic lifestyle of *L. pneumophila*, several T4ASSs, of either F or P subtypes, or both, have also been identified in the genome of each strain sequenced [22]–[24]. The *Legionella* F-type T4ASSs, known as the Lvh and Tra regions, have both been found either as extrachromosomal, plasmid-like elements, or integrated into the chromosome in some strains [22], [25]. In other strains, there are multiple copies of the same element, for example, two Lvh-regions are integrated into the chromosome of strain 130b [24]. These chromosome-integrated T4SSs are associated with factors usually found encoded on mobile genetic elements such as integrase genes, regulatory genes and flanking repeats, which suggests they could also be mobilised and may be activated during certain stages of the organism's life cycle [22]. There is evidence that components of the T4A and T4BSSs, which are encoded in different locations on the genome, are able to interact with one another, as the Lvh-region has been demonstrated to restore some functions of the Dot/Icm system in deficient mutants [26], [27]. This, together with the fact that multiple T4SSs have been found per *Legionella* genome, suggests that all these secretion systems play an important role in the biology and evolution of this organism.

In addition to these T4A and T4BSSs, we recently identified two new putative T4SSs in *L. pneumophila* genomes with similarity to GI-T4SSs, which were collectively named *Legionella* GI-T4SS 1 and *Legionella* GI-T4SS 2 [24]. A recent study showed that the two GI-T4SSs in *L. pneumophila* strain Corby are associated with genomic islands (LpcGI-1 and LpcGI-2) capable of excising from the chromosome and circularising. The authors also provide experimental evidence that LpcGI-2 is a functional ICE [28]. Here, we investigate the distribution and conservation of GI-T4SS genes in publically available *Legionella* genomes and describe the features of the genomic loci in which they are found. We show that the *Legionella* GI-T4SSs are well conserved in almost all members of this genus, and belong to a distinct lineage that is divergent from other GI-T4SS.

## Materials and Methods

### Bacterial genomes

The complete genome sequences of *Legionella pneumophila* serogroup 1 strains Alcoy, Corby, Philadelphia 1 (Phila), Paris and Lens, together with *L. longbeachae* strain NSW150 were used in this study [20], [23], [29]–[31]. Three draft genome sequences, of *L. pneumophila* serogroup 1 strain 130b, *L. longbeachae* D-4968 and *L. drancourtii* LLAP12, were also included in the analyses [24], [32], [33] (Table 1).

**Table 1:** Distribution of GI-T4SSs in *Legionella*.

Species	Strain	Accession number and status	Ref.	LGI	Locus tags – LGI	Insertion site (*attL*)	Size* (Kb)	Direct repeats$ (*attR*)	Locus tags – LGI-T4SS
*L. pneumophila*	Alcoy	CP001828 (Complete)	[20]	LpaGI-1	lpa_01465 - lpa_01688	tRNA^Thr^ (lpa_05026)	51	43 (6M), 40 (10M)	lpa_01534 - lpa_01501
	Corby	CP000675 (Complete)	[23]	LpcGI-1	LPC_2314 - LPC_2170	tRNA^Thr^ (LPC_2315)	45	43 (7M), 37 (9M)	LPC_2273 - LPC_2296
				LpcGI-2	LPC_1833 - LPC_1888, LPC_2136 - LPC_2121	tRNA^Met^ (LPC_1832)	22	45 (0M), 48 (2M)	LPC_1857 - LPC_1880
	Philadelphia 1	AE017354 (Complete)	[31]	LpgGI-1	lpg0973 - lpg1085	tRNA^Thr^ (lpg0972)	115	42 (0M), 43 (6M)	lpg0983 - lpg1005
	Paris	CR628336 (Complete)	[30]	LppGI-1	lpp1034 - lpp1088	tRNA^Thr^	45	43 (7M)	lpp1075 - lpp1055
				LppGI-2	lpp2311 - lpp2439	tRNA^Met^	123	47 (0M), 45 (4M)	lpp2375 - lpp2398
	Lens	CR628337 (Complete)	[30]	LplGI-1	lpl1001 - lpl1085	tRNA^Thr^	80	43 (0M), 43 (0M)	lpl1016 - lpl1039
	130b	CAFM00000000 (Draft, 159 contigs)	[24]	LpwGI-1	LPW_10581- LPW_11301	tRNA^Thr^	59	43 (0M)	LPW_10961 - LPW_10731
				LpwGI-2	LPW_21181- LPW_22131	tRNA^Arg^	79	48 (0M)	LPW_21631 - LPW_21861
*L. longbeachae*	NSW150	FN650140 (Complete)	[29]	None	-	-	-	-	-
	D-4968	ACZG00000000 (Draft, 13 contigs)	[33]	LlbGI	LLB_1155 - LLB_1201	tRNA^Pro^ (LLB_1202)	>45	-	LLB_1169 - LLB_1191
*L. drancourtii*	LLAP12	ACUL00000000 (Draft, 263 contigs)	[32]	LdrGI-a	LDG_0891 - LDG_0943	tRNA^Arg^ (LDG_3797)	>46	-	LDG_0920 - LDG_0896
				LdrGI-b	LDG_2365 - LDG_2398	tRNA^Pro^ (LDG_3807)	>30	-	LDG_2375 - LDG_2397
				LdrGI-c	LDG_2949 - LDG_3002	unknown	>44	-	LDG_2995 - LDG_2965
				LdrGI-d	LDG_3725 - LDG_3784	unknown	>58	-	LDG_3747 - LDG_3747

* Size from *attL* to last *attR.*

$ Number of base pairs in direct repeat of *attL*: *attR1* (no. mismatches), *attR2* (no. mismatches).

### Annotation and comparative genomics

Predicted coding sequences (CDSs) within and surrounding the LGI regions in the annotated genomes were further investigated by searching against the Uniprot protein sequence database using FASTA [34]. Conserved domains and protein families were identified using Interpro Scan and CD-Search [35], [36]. Transmembrane helices, signal peptides and protein localization signals were classified using TMHMM v2.0, SignalP v3.0 and PSORT programs [37]–[39]. Manual curation of the annotation was facilitated using Artemis [40]. Repeated sequences delineating the putative genomic islands were identified using a self-against-self BLASTN [41] nucleotide similarity search with a seed length of seven and an e-value cutoff of 10, to allow detection of short repeats of at least 7 bps.

Whole genome alignments generated by BLASTN were visualized using the Artemis Comparison Tool [42]. Pairwise average nucleotide identities (ANI) were calculated from BLASTN matches as implemented in JSpecies v.1.2.1 [43] with default settings. To find elements closely related to the LGIs, TBLASTX and Position-Specific Iterative (PSI)-BLAST (until the results converged or a maximum of 5 iterations was reached) searches of the NCBI nucleotide (nt) and non-redundant (nr) protein databases respectively were performed. Hits were searched for matches to the well-characterised T4SS genes of the *E. coli* F-plasmid and the *A. tumefaciens* VirB/D4 system and pairwise comparisons of the LGIs were visualised using Easyfig [44]. All comparative genomic figures were prepared using Easyfig.

### Phylogenetic analyses

To examine the phylogeny of the LGI-T4SS clusters, nucleotide sequences of 15 *lgi* genes (*lgiA*, *lgiB*, *lgiC*, *lgiD*, *lgiE*, *lgiF*, *lgiG*, *lgiH*, *lgiI*, *lgiJ*, *lgiL*, *lgiO*, *lgiP*, *lgiQ* and *lgiT*) from 14 LGI-T4SS clusters were aligned using clustalW2 [45]. Five *lgi* genes were not used because they were either not as well conserved and resulted in poor alignments (*lgiM, lgiR, lgiS*), or had disrupted CDSs (pseudogenes) in some strains (*lgiN* and *lgiK*). A maximum likelihood (ML) tree was constructed for each gene alignment using the General Time Reversible (GTR) model with an estimated gamma distribution with 4 rate categories and an estimated proportion of invariable sites, using PhyML [46]. These 15 trees were visualized as a Consensus Network using SplitsTree (v 4.10) [47].

To determine the evolutionary relationship between the LGI-T4SS and other T4SSs, the amino acid sequences of VirB4/TraC/LgiN homologues were taken from 13 LGIs (all except LdrGI-a which had a disrupted *lgiN*) as well as from representatives of the major T4SS types, namely *A. tumefaciens* plasmid Ti (NC_002377), *E. coli* F plasmid (NC_002483), IncP-alpha plasmid (NC_001621), Plasmid R64 (AB027308), *H. influenzae* plasmid ICE*Hin*1056 (NC_011409), *P. aeruginosa* PAPI-1 (AY273869), *P. aeruginosa* strain C plasmid pKLC102 (AY257538), and *S. bongori* ICE*Sb*1 (FN298494). Sequences were aligned using ClustalW2 and a ML tree was constructed with the WAG amino-acid substitution model using FastTree 2.1.4 [48]. 1000 bootstrap replicates were made to evaluate support for each clade.

### Recombination

The RDP package (v4.22) [49] was used to identify recombination in the LGI-T4SSs from the concatenated sequences of the 15 conserved *lgi* genes (*lgiA*, *lgiB*, *lgiC*, *lgiD*, *lgiE*, *lgiF*, *lgiG*, *lgiH*, *lgiI*, *lgiJ*, *lgiL*, *lgiO*, *lgiP*, *lgiQ* and *lgiT*) that were also used to build the LGI-T4SS phylogeny, as described above. Analyses were performed on the concatenated sequences to allow us to detect recombination events that involved multiple contiguous genes. Recombination events were deemed significant only if they were detected by at least two out of the six methods (RDP, GENECONV, BootScan, MaxChi, Chimaera, Siscan). Default settings for each method were used, including a P-value cutoff of 0.05 with the Bonferroni method to correct for multiple testing.

### Selection analysis

Alignments of each of the 15 representative *lgi* genes (listed above) from each of 12 LGI-T4SS clusters (LdrGI-c and LdrGI-d were excluded from this analysis as they were too divergent to compare selection signals reliably) were made at the codon level using ClustalW2 as implemented in MEGA [50]. Stop codons were removed from the ends of each sequence and a Neighbour-Joining tree constructed for each gene alignment using ClustalW2 [45].

The ratio of non-synonymous substitutions per non-synonymous site and synonymous substitutions per synonymous site (dN/dS) was estimated using branch models implemented by CODEML in the Phylogenetic Analysis by Maximum Likelihood (PAML) v4.4. package [51]. Model = 0 (one dN/dS ratio for all branches) and model = 2 (branches are assigned to one of two dN/dS ratio classes) were used to estimate dN/dS values, and maximum likelihood values were calculated for each model. A likelihood ratio test (LRT), comparing twice the difference in log likelihoods, was then performed for each pair of nested models in order to test a number of hypotheses, as described below.

## Results and Discussion

### The *Legionella* GI-T4SS is a conserved cluster of genes associated with conjugation

**Figure 1:**
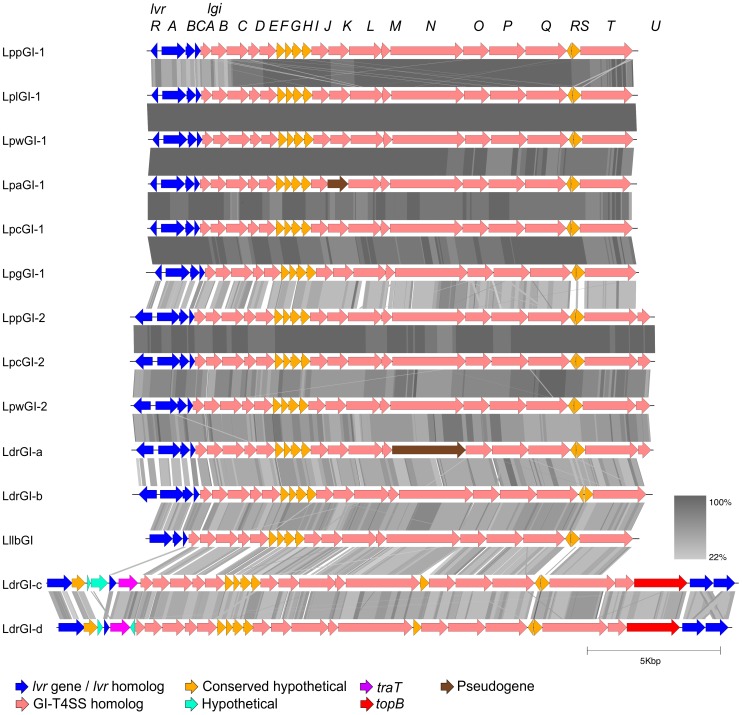
Genetic organisation of the 14 LGI-T4SS clusters in *Legionella*. Each of the 14 *Legionella* GI-T4SS (LGI-T4SS) clusters found in the genomes of sequenced *L. pneumophila*, *L. drancourtii* and *L. longbeachae* strains are presented, and labeled according to the LGI on which they are located. Common to all LGI-T4SSs are a highly conserved cluster of 20 *lgi* genes (*lgiA-T*), and regulatory *lvr* genes. CDSs are represented as arrows, coloured according to their function as described in the key. The translated nucleotide sequence identity (tBLASTx) between each cluster is represented by grey bars, shaded according to the percentage of amino acid identity as shown in the key.

The *Legionella* GI-T4SS (LGI-T4SS) gene cluster was found to be present in eight of the nine *Legionella* spp. genome sequences that were publically available at the commencement of this study (see Table 1). Although no LGI-T4SS cluster was identified in the genome sequence of *L. longbeachae* NSW150, the other eight genomes were each found to encode between one and four distinct LGI-T4SSs. In total, 14 loci were investigated and assigned unique identifiers (Table 1). The LGI-T4SS cluster consists of 24 genes, designated *lvrRABC* and *lgiA-T* (Figure 1 and Table S1) [24].

At the 5′ end of the cluster, the first four genes (*lvrRABC*) form a regulatory module unique to *Legionella*. The gene *lvrR* encodes domains that are homologous to those involved in the regulation of SOS-response genes in *Escherichia coli* [52] while *lvrC* encodes a protein containing a putative carbon storage regulator domain (CsrA) shown to play a role in the regulation of gene expression during certain growth stages of the organism [22], [53]. These *Legionella* Vir Region (lvr) genes are so named because they were first found flanking the *Legionella* vir homologue (lvh), a *Legionella* T4ASS homologous to the Vir system in *Agrobacterium tumefaciens*. Copies of the *lvr* genes were also identified upstream of other T4ASSs, both P- and F-type, in *Legionella* [22], [24]. The association of the *lvrRABC* genes with multiple T4SSs in *Legionella* suggests a role in coordinating the expression of the T4SS, and these genes were recently shown to regulate the excision of the genomic island encoding the T4ASS Trb-1 in strain Corby [28].

**Figure 2:**
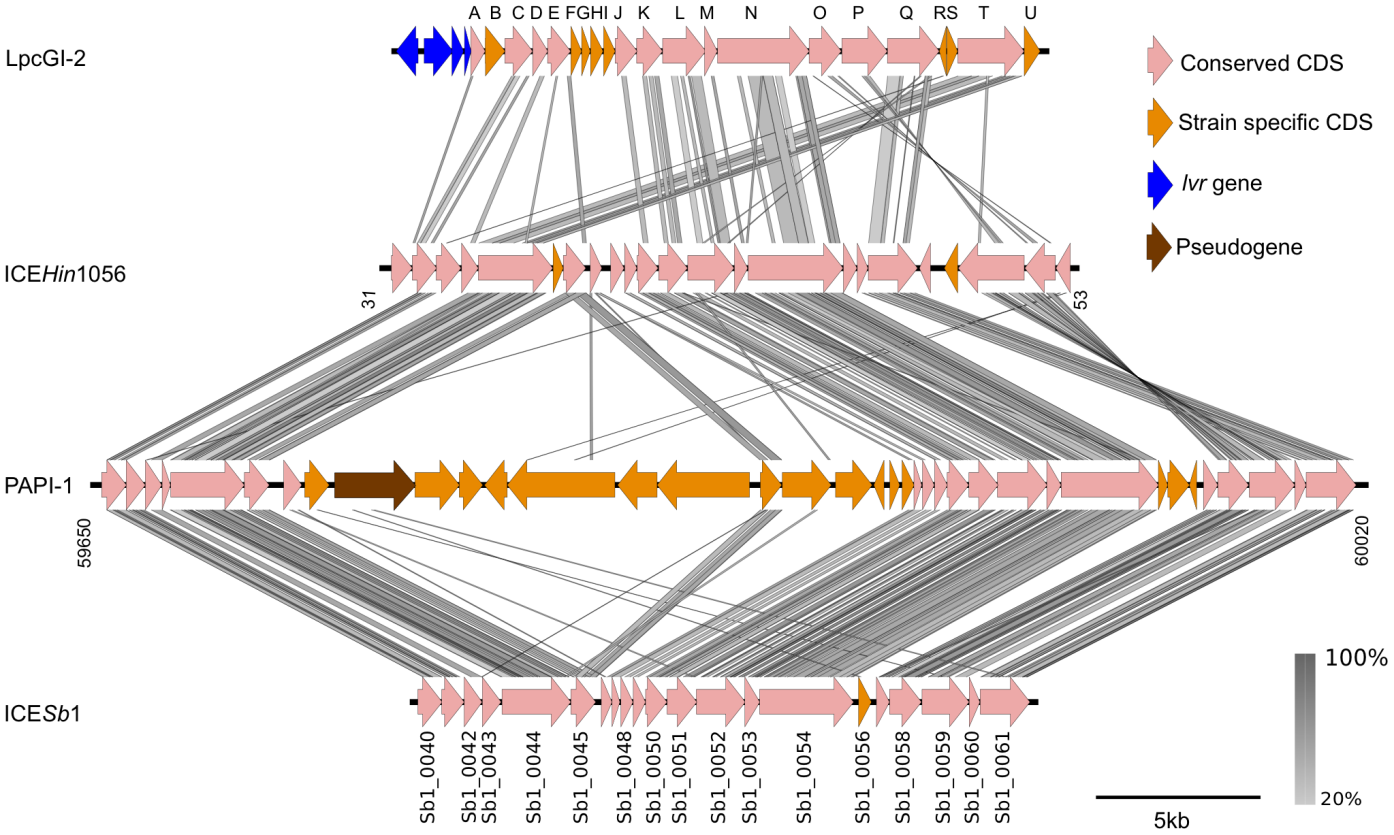
Comparison between GI-T4SS clusters in *Legionella* and SPI-7 family ICEs. LpcGI-2 from *L. pneumophila* strain Corby is representative of the conserved LGI cluster in *Legionella* spp. and compared to the well-characterised SPI-7 family ICEs from *Haemophilus influenzae* (ICE*Hin*1056), *Pseudomonas aeruginosa* (PAPI-1) and *Salmonella bongori* (ICE*Sb*1). Similarity between the different GI-T4SS clusters is represented by grey bars, shaded according to the percentage of amino acid identity as shown in the key. Genes are coloured according to category as described in the key.

A continuous stretch of 20 genes (*lgiA-T*) is well conserved across all 14 LGI-T4SS clusters (Figure 1 and Table S1). Thirteen of these genes are homologous to genes found in other previously characterised GI-T4SS such as those in *Haemophilus influenzae* (ICE*Hin*1056), *Pseudomonas aeruginosa* (PAPI-1) and *Salmonella bongori* (ICE*Sb*1), as described in Table 2, and the gene order is largely conserved (Figure 2). The sequence similarity between the different GI-T4SSs is detectable only at the amino acid level, suggesting that these systems are separated by long evolutionary distances, as is shown from the translated nucleotide BLAST comparison in Figure 2. All thirteen genes were shown to be essential for conjugation of the associated mobile element in at least one of these other organisms [6], [11], [18]. Additionally, *lgiP* (*pilT*) was recently shown to be necessary for the conjugation of the associated genomic island in *L. pneumophila* strain Corby [28]. The remaining seven *lgi* genes (*lgiB*, *lgiF*, *lgiG*, *lgiH*, *lgiI*, *lgiR* and *lgiS*) did not have any significant similarity to other sequences in the public databases.

**Table 2:** ICE core genes in ICE*Hin*1056, ICE*Sb*1 and PAPI-1 and corresponding LGI homologues.

LGI	ICE*Hin*1056 ^[19]^	ICE*Sb*1 ^[11]^	PAPI-1 ^[18]^	Gene name	Putative function/Description
Replication					
	p1056.01	Sb0003**	PA14_58910**	*soj/parA*	ATPase. Chromosome partitioning
	p1056.02	Sb0004**	PA14_58990**	*dnaB*	Helicase
	p1056.03	Sb0005**	PA14_59070**	*parB*	Nuclease
	p1056.04	Sb0009**	PA14_59090**		
	p1056.05	Sb0011**	PA14_59100**		
	p1056.06	Sb0012**	PA14_59130**		
	p1056.07	-	PA14_59140**		
α	p1056.08	Sb0016	PA14_59150	*ssb*	Single-stranded DNA binding
β	p1056.11	Sb0013*	PA14_59180	*topB*	Topoisomerase
	p1056.12	Sb0014*	PA14_59400		
	p1056.14	-	-	*radC*	DNA repair
Conjugation (GI-T4SS)					
*lgiA*	p1056.31**	Sb0018**	PA14_59240**	*pilL*	Membrane bound lipoprotein
*lgiC*	p1056.32	Sb0040**	PA14_59650**	*traG*	
*-*	p1056.33*			*traL*	
*-*		Sb0041**	PA14_59660**		
*lgiD*	p1056.34	Sb0042**	PA14_59670**	*traW*	
			PA14_59680**		
*lgiT*	p1056.35**	Sb0044**	PA14_59690**	*traD/VirD4*	ATPase. Coupling protein
*lgiE*	p1056.37	Sb0045**	PA14_59700**		
	p1056.38	Sb0046**	PA14_59870**		
		Sb0047**			
		Sb0048**	PA14_59880**		
	p1056.40	Sb0049**	PA14_59890**		
*lgiJ*	p1056.41**	Sb0050**	PA14_59900**	*dnaA*	
*lgiK*	p1056.42	Sb0051**	PA14_59910**		
*lgiL*	p1056.43**	Sb0052**	PA14_59920**	*traB/virB10*	Part of T4SS pore complex
*lgiM*	p1056.44	Sb0053**	PA14_59930**		
*lgiN*	p1056.45**	Sb0054**	PA14_59940**	*traC/virB4*	ATPase. Energy for T4SS assembly
	p1056.47	Sb0060∧	PA14_60010**		
*lgiQ*	p1056.48	Sb0061**	PA14_60020**	*traG*	Mating pore stabilization
	p1059.49	Sb0062**	-		
*lgiP*	p1056.51**	Sb0059∧	PA14_60000**	*pilT*	
*lgiO*	p1056.52**	Sb0058**	PA14_59990**	*traU*	Mating pore stabilization
	p1056.53*	Sb0057**	PA14_59980**		
Integration					
	p1056.62	Sb0117**	PA14_60130**	*traI*	Relaxase
?	p1056.63	Sb0118**	PA14_60140**	*int*	Integrase

**α** *ssb* is only present near LGI-1 clusters in *L. pneumophila* strains and LdrGI-c.

**β** *topB* is only present near LdrGI-c and LdrGI-d.

**χ** all LGIs have integrases unrelated to those in ICE*Hin*1056, ICE*Sb*1 and PAPI-1.

** Essential for conjugation.

* Non-essential but deficiency significantly reduces conjugation.

∧ Not required for conjugation.

Of the 13 *lgi* genes that were homologous to those in other GI-T4SS, only nine (*lgiA*, *lgiC lgiD*, *lgiL lgiN*, *lgiO*, *lgiP*, *lgiQ* and *lgiT*) shared some sequence similarity with genes encoding well-characterised T4SS subunits, namely PilL, TraG, TraW, TraB, TraC, TraU, PilT, TraG and TraD, respectively [6], [11]. These nine subunits play important roles in the formation and stabilisation of the conjugative pilus, as well as providing an energy source, via ATPases, for the assembly of the T4SS complex (Table 2). Only one subunit, VirB4/TraC, is ubiquitous in all T4SS found to date [1], [54].

### LGI-T4SS clusters are conserved across multiple loci within hypervariable genomic islands

The LGI-T4SSs are all located on genomic islands, which we refer to henceforth as *Legionella* Genomic Islands (LGI). Flanking the LGI-T4SS clusters within each of the LGIs are variable genomic regions that indicate multiple insertion, deletion and translocation events, typical of genomic islands and other mobile genetic elements (Figures S1 and S2). The LGIs are all found within hypervariable regions of the genome, adjacent to tRNA genes, which are typical recognition sites for site-specific recombinases such as integrases and transposases [55], suggesting that these regions are hotspots for the acquisition of horizontally acquired DNA. Indeed there are several direct repeats flanking the LGIs, which correspond to the last 37 to 48 bp at the 3′ end of the tRNA genes adjacent to the LGIs (Table 1, Figures S1 and S2). These repeats indicate that there have been multiple insertion events in these regions, as such repeats occur when a mobile element is inserted via site-specific recombination into a conserved chromosomal sequence (*attB*) that is also present on the mobile element (*attP*). The resulting insertion is bounded by hybrid insertion sequences *attL* and *attR* that appear as direct repeats of *attB* [56]. The direct repeats flanking the LGI were recently shown to correspond to *attL* (tRNA 3′ end) and *attR* (3′ end direct repeats) sites in strain Corby (see Figures S1 and S2), with each *attR* associated with separate mobile elements, each capable of excision and circularisation [28].

The members of LGI-1 are all found adjacent to a tRNA^Thr^ gene in a syntenic genomic location in all six *L. pneumophila* genomes studied (Table 1 and Figure S1). The GI-T4SSs on each of the six LGI-1s share 89–100% pairwise Average Nucleotide Identity (ANI) with each other, which is comparable to the 96–99% ANI shared between the whole genomes of these strains (Tables S2 and S3).

In addition to LGI-1, the genomes of *L. pneumophila* strains Paris, Corby and 130b also encode a second LGI (LGI-2) elsewhere in their genomes. LGI-2 in Paris (LppGI-2) and Corby (LpcGI-2) are found in syntenic positions, adjacent to the same tRNA^Met^ gene, and their GI-T4SSs share 97% ANI. LGI-2 in strain 130b (LpwGI-2), is found in a different genomic location, adjacent to a tRNA^Arg^ gene, and its T4SS has only 85% ANI to those of LppGI-2 and LpcGI-2 (Table 1 and Table S2). LpwGI-1 and LpwGI-2 are each located within scaffolds, with paired 454 reads confirming their genomic locations, in the draft genome of 130b.

Although we did not identify any LGI-T4SS clusters in the genome sequence of *L. longbeachae* strain NSW150, the draft genome of *L. longbeachae* D-4968 contains a single cluster, LlbGI, located on one of two main contigs that cover more than 60% of the genome. In addition, *L. drancourtii* LLAP12 encodes four distinct clusters: LdrGI-a, LdrGI-b, LdrGI-c and LdrGI-d (Table 1). These four *L. drancourtii* LGI-T4SS clusters are found on separate contigs that are between 43 and 59 kb in length [32]. The maximum ANI between any two of the *L. drancourtii* clusters is below 80% (Table S2) and the sequences flanking the clusters were highly divergent, indicating that they are unlikely to have been misassembled. However, due to the fragmented nature of the *L. drancourtii* LLAP12 draft genome coupled with the high frequency of genomic shuffling evident in *Legionella* genomes, the exact genomic positions of the four *L. drancourtii* clusters is uncertain, although we could ascertain that LdrGI-a, like LpwGI-2, is adjacent to a tRNA^Arg^ gene, and LdrGI-b and LlbGI are both associated with tRNA^Pro^ genes.

The LGI-T4SSs are largely conserved in gene order and composition between the different *Legionella* strains, however the GI-T4SS of *L. longbeachae* and two of the *L. drancourtii* LGI-T4SSs are missing parts of the usual *lvrRABC* regulatory operon at the 5′ end of clusters (Figure 1). LlbGI is missing *lvrR*, and although the other three genes, *lvrABC*, were present, no other gene with a regulatory domain was found associated with this LGI-T4SS cluster. Instead of the *lvrRABC* operon, LdrGI-c and LdrGI-d both have two genes that share low sequence identity to *lvrA* and *lvrC* together with a gene homologous to the F-plasmid entry exclusion gene *traT* (Figure 1). At the 3′ end of the GI-T4SSs on both LdrGI-c and LdrGI-d is a gene encoding topoisomerase (*topB*) and two copies of *lvrE*, a gene that is also found 3′ of the Lvh T4ASS in *L. pneumophila* [24], [30], [31]. This peculiar arrangement of *lvr* genes suggests that the T4SSs in LlbGI, LdrGI-c and LdrGI-d may be regulated differently from the other LGI-T4SS clusters.

In addition to the *lvr* gene rearrangements, *lgi* genes in two LGI-T4SS clusters were found to be disrupted and presumed to be pseudogenes (Figure 1). The *lgiK* gene in LpaGI-1 is truncated by a premature stop codon, and the *lgiN* gene in LdrGI-a contains a frameshift mutation, however this is within a homopolymeric tract and thus may have been caused by a pyrosequencing error based on the technology used to sequence this genome.

### The 14 LGI-T4SSs form three distinct phylogenetic clades

**Figure 3:**
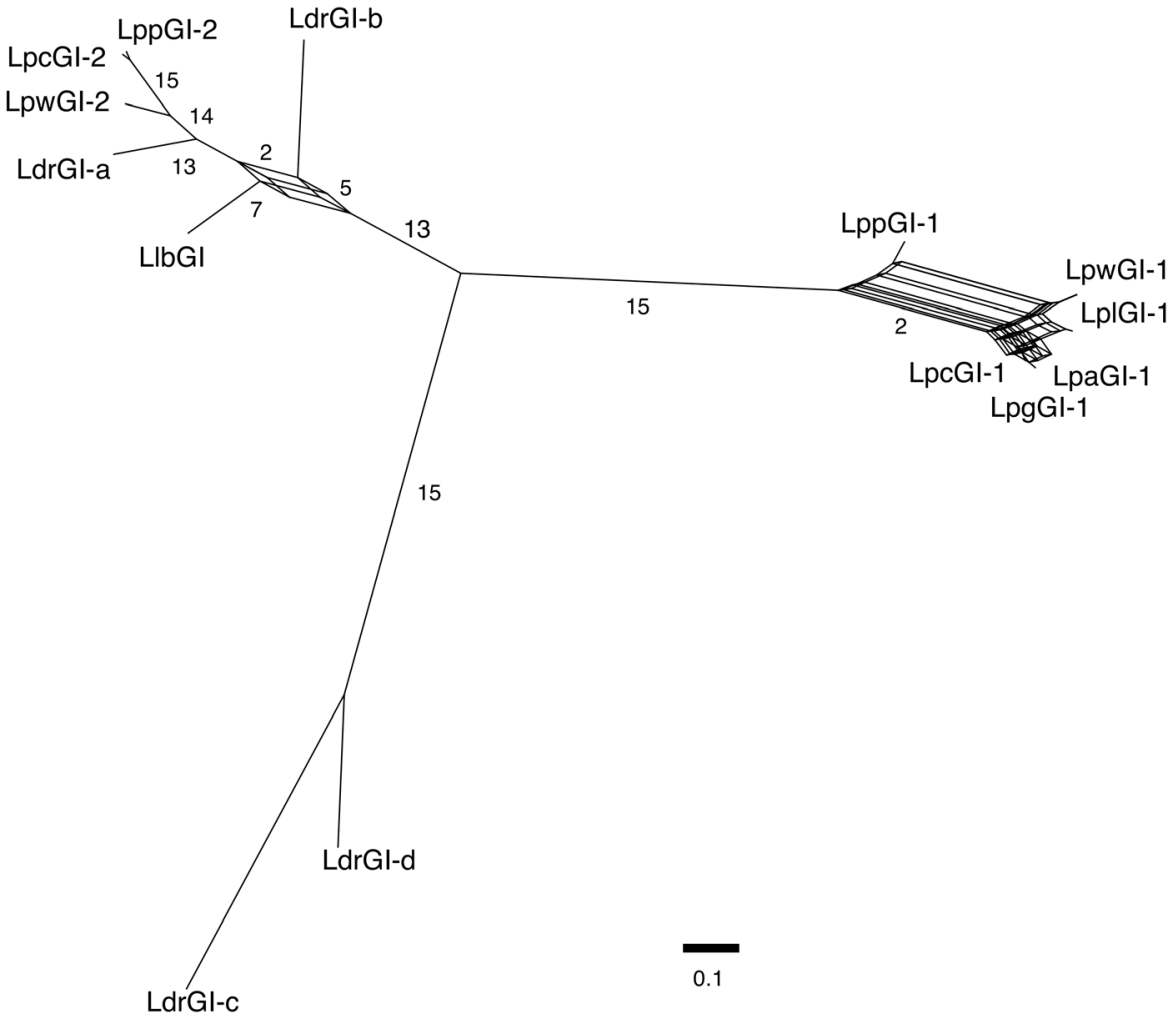
Phylogenetic relationship between LGI-T4SS clusters. A consensus network tree showing the relationship between the 14 *Legionella* GI-T4SS clusters, constructed from the individual maximum likelihood trees of 15 conserved *lgi* genes. Branch labels indicate the number of trees that support each branch. Branch lengths show the number of substitutions per site, as indicated by the scale bar, and are averaged across the trees that contain that branch.

To determine the relationships between the 14 LGI-T4SSs, a phylogenetic analysis was carried out using alignments of 15 genes that were conserved in all 14 clusters (*lgiA, lgiB, lgiC, lgiD, lgiE, lgiF, lgiG, lgiH, lgiI, lgiJ, lgiL, lgiO, lgiP, lgiQ, lgiT*). The four *lvr* genes and five *lgi* genes not included in this analysis were genes that in some clusters were missing, disrupted, shorter than 400 bp, or less conserved (see Figure 1), resulting in poor alignments. A consensus network was constructed from the 15 individual maximum likelihood phylogenetic trees generated, which confirmed that the syntenic LGI-1 T4SS clusters form a very closely related group (Figure 3). Thirteen of the 15 genes supported the groupings of the LGI-T4SS clusters into three main clades, with the LGI-1s in the first, LdrGI-c and LdrGI-d in the second and the rest in the third clade (Figure 3). The most striking feature in this network representation is the high level of phylogenetic incongruence within the LGI-1 clade, which indicates that extensive recombination events have occurred between the LGI-1 T4SSs.

There is substantially less phylogenetic incongruence in clade three, with only two genes, *lgiI* and *lgiT*, showing evidence of disagreement about the placement of LlbGI and LdrGIb. Despite the generally consistent phylogenetic signal of the genes in these clusters, however, the disparate genomic locations of the clusters suggests that this group of LGI-T4SSs have undergone transpositions, duplications, and/or undetected recombination events. Thus, the numbering system used in this study for the non-LGI-1 clusters is solely based on the number of clusters in each genome and does not denote orthology.

To investigate the underlying causes of the ambiguities in the phylogeny of the LGI-T4SS clusters, we performed recombination analyses on the concatenated 15 gene alignment used to build the phylogenetic network. Using a combination of six methods implemented in the RDP software package, we identified several recombination events that were likely to have occurred, based on agreement of at least two out of the six methods used. All of the large recombination events, larger than 500 bp in size, occurred only within the LGI-1 clade (Table S4, Figure S3), which explains the ambiguous topology of that clade in the phylogenetic network (Figure 3). Higher levels of recombination were found between more closely related LGI clusters, most likely due to the higher chances of homologous recombination occurring. Recombination between the major clades was not detected. *Legionella* has an exceptionally high level of genome flux which results in a *L. pneumophila* species tree that is notoriously difficult to resolve beyond the established close relationships between strains Lens and 130b, and that between Alcoy and Corby [20], [24]. Nevertheless, these results suggest that the LGI-1 elements were present in the last common *L. pneumophila* ancestor and that the major LGI-T4SS clades are likely to have diverged before the last common ancestor of the *Legionella* strains in this study.

LdrGI-c and LdrGI-d form a lineage that is clearly divergent from the other LGI-T4SS clusters. These two divergent clusters in *L. drancourtii* shared only a maximum of 67% ANI with any of the other 12 clusters and were similarly divergent from each other (Table S2). This level of divergence is close to the whole genome divergence between *L. drancourtii*, *L. pneumophila* and *L. longbeachae* (72%–75% ANI, Table S3) suggesting that these two clusters may have diverged before the last common ancestor of these three *Legionella* species.

### The LGI-T4SS represents a distinct and novel lineage of GI-T4SS

To determine the evolutionary relationship between the LGI-T4SS clusters and other T4SSs, a phylogenetic analysis was performed on VirB4/TraC, the only protein encoded in all lineages of T4SSs. Sequences of TraC (F plasmid), TrbE (IncP), VirB4 (pTi, *A. tumefaciens*) and TrbU (R64) were included in the analysis to represent the three main T4SS subgroups. GI-T4SS was represented by the VirB4/TraC homologue from ICE*Hin*1056, PAPI-1, ICE*Sb*1, and pKLC102. Complete LgiN sequences from 13 LGI-T4SS clusters (all except LdrGI-a which had a truncated *lgiN* gene) were used as the VirB4/TraC homologue to represent the LGI-T4SSs.

**Figure 4:**
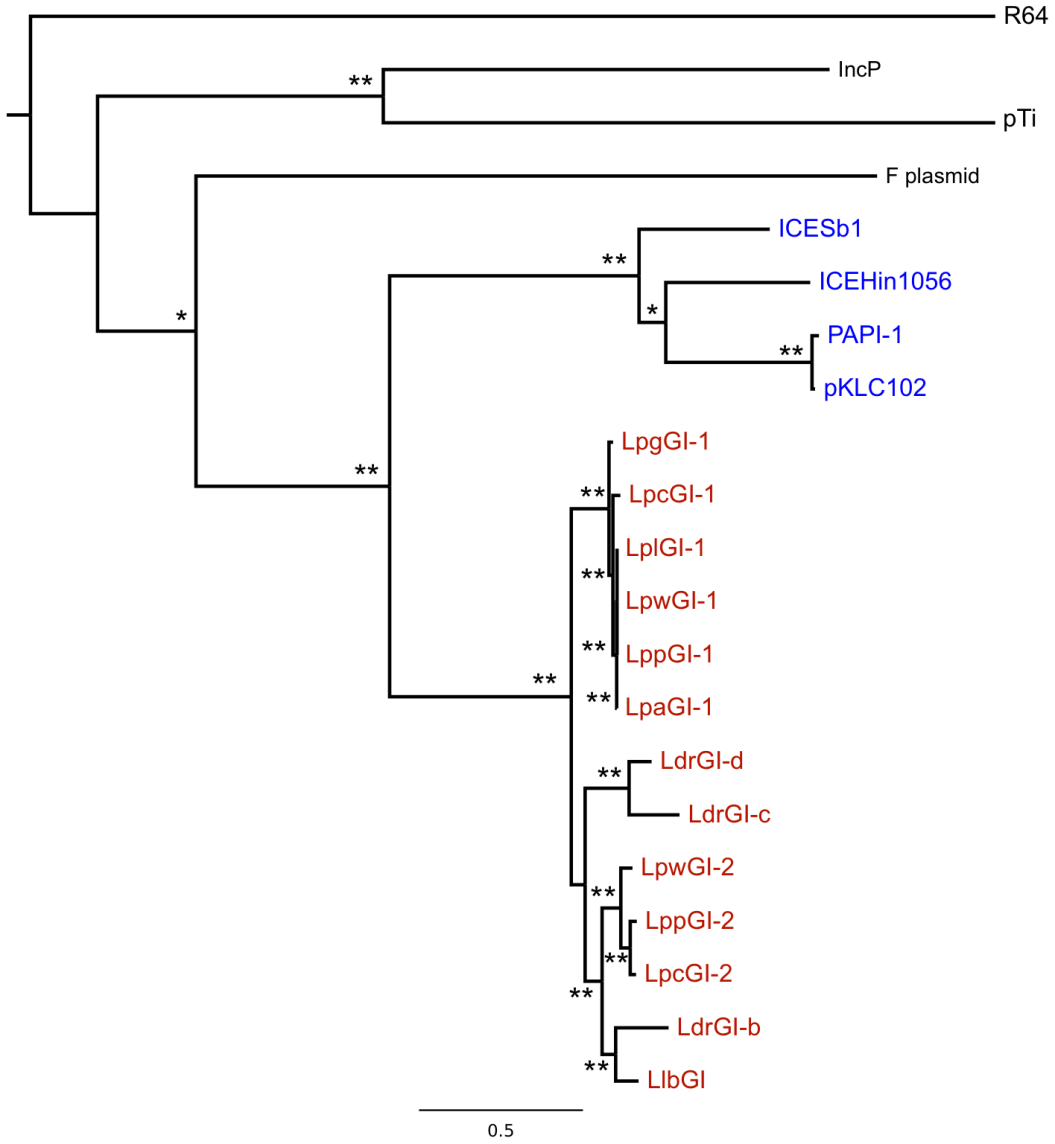
Phylogeny of GI-T4SS across representative Proteobacterial genomes. Maximum likelihood tree of LgiN/VirB4/TraC homologues showing the divergent *Legionella* GI-T4SS clade (red). Representatives from the SPI-7 family of ICE are highlighted in blue, with representatives of the T4ASS (*A. tumefaciens* plasmid Ti, *E. coli* F plasmid and IncP-alpha plasmid) and T4BSS (Plasmid R64) in black. Asterisks indicate branches with percentage support from 1000 bootstrap replicates above 80% (** >90%, * >80%).

The resulting phylogenetic tree (Figure 4) supports the previous grouping of the GI-T4SS clade as separate from the T4A and T4B systems [57]. Within the GI-T4SS clade, the LGI-T4SSs form a monophyletic group divergent from other GI-T4SSs. This separation of clades is supported by more than 99% of the bootstrap tests and was also replicated in extended phylogenies of GI-T4SS homologues across alpha, beta and gamma-Proteobacteria genomes (data not shown). This phylogenetic signal is consistent with the other observations presented in this study suggesting that the LGI-T4SS is indeed a novel and divergent lineage of the GI-T4SS.

### The LGI-T4SS is under purifying selection pressure

The selection pressure acting on a horizontally acquired DNA fragment is determined by how it contributes to the fitness of the organism, or element, that acquires it. If the genes encoded by this DNA are beneficial, amino acid-changing mutations may accumulate in them more slowly than if they provide no selective advantage. Ultimately, dispensable genes may either be degraded or entirely deleted from the genome due to the effect of a deletional bias present in bacterial genomes [58]. The conservation of the LGI-T4SS cluster as a predominantly intact stretch of at least 20 genes would suggest that it is actively playing an important role in *Legionella*. To further confirm that these genes are being maintained by selection, rather than evolving neutrally, we ran selection analyses on alignments of the 15 conserved LGI-T4SS genes (described above), estimating the ratio of the rate of non-synonymous substitutions per non-synonymous site (dN) to the rate of synonymous substitutions per synonymous site (dS) for each gene. Genes that are evolving neutrally, suggesting that they are not beneficial to *Legionella* and may be degraded, are expected to have a dN/dS ratio of one or close to one and genes that are evolving under purifying selection, suggesting they are beneficial, should have a dN/dS ratio of significantly less than one [59]. Selection analyses were carried out despite the recombination events detected in the LGI-1 clade, as the presence of recombination may artefactually increase the dN/dS ratio making it less likely to reject the null hypothesis of neutral evolution, thus making the test more conservative [60].

First, we tested whether the dN/dS values for these genes were significantly different between the LGI-1 clade and the other LGI-T4SSs. It is reasonable to assume that the more closely related LGI-1 GI-T4SSs are orthologous, and they may thus all play a similar biological role, while the other LGI-T4SSs are paralogous and may have evolved a different function [61]. Therefore, we tested whether the T4SS genes are under stronger purifying selection in LGI-1 than in other LGIs. For each gene, the dN/dS values for the LGI-1 clade and the other LGI-T4SSs (LppGI-2, LpcGI-2, LpwGI-2, LdrGI-a, LdrGI-b and LlbGI) were compared. LdrGI-c and LdrGI-d were not included in this analysis because they do not obviously cluster with either of the main clades and may be evolving under different constraints from both of them. For nine of the genes (*lgiABCDEFGHI*), dN/dS values were not significantly different between T4SSs from LGI-1 and non-LGI-1 clades. The remaining genes (*lgiJLOPQR*), however, had significantly lower dN/dS values in LGI-1 than in the other LGI-T4SSs (p<0.01). The lower dN/dS of *lgiJLOPQR* in LGI-1 may be indicative of stronger purifying selection acting on LGI-1 T4SSs. Alternatively, it could also mean that the more diverse non-LGI-1 clade has a larger range of dN/dS caused by weaker purifying selection acting on some of them. The absence of significant difference in dN/dS for *lgiABCDEFGHI* may be due to gene length, as these genes are all shorter than 300 bp, while the *lgiJLOPQR* genes, which show significant differences, are all longer. More sites in these longer genes give us more power to reject the hypothesis of a single dN/dS value between the two clades being tested. Nevertheless, all 15 genes in both clades have a dN/dS that is significantly less than 1 (p<0.01) (Table S5).

Secondly, we tested specifically whether the presence of two LGI-T4SS clusters in a genome affected the selection acting on each version. In those genomes in which a second cluster (on LGI-2) was present in addition to the LGI-1 T4SS, we might expect the T4SS in either LGI-1 or LGI-2 to be under purifying selection and the other to be evolving neutrally if only one was required. To test this, we compared the dN/dS of LGI-1 T4SS genes to the dN/dS values of the second LGI-T4SSs in each genome (those in LpcGI-2, LppGI-2 and LpwGI-2). LGI-T4SSs from *L. drancourtii* LLAP12 were not tested as there were four clusters in total and only one cluster in *L. longbeachae* D-4968. We found that the T4SSs from LGI-1 and LGI-2 clades were evolving under similar purifying selection pressures, as the differences in dN/dS values between these two clades were not significant for 13 of the 15 genes tested; all 15 genes had dN/dS values that were significantly less than 1 (p<0.01) (Table S5). These results suggest that all LGI-T4SS clusters in the genome play important roles, rather than being redundant.

The dN/dS values of all tested LGI-T4SS genes are significantly less than one, indicating that these genes are under purifying selection pressure. These values are close to those found in a previous study that measured the dN/dS of several core *Legionella* genes as well as a number of genes that were predicted to be of eukaryotic origin and thus important to the parasitic lifestyle of *Legionella* [62].

### The LGI is not a typical ICE

Characterised GI-T4SSs have so far been exclusively associated with a family of ICEs that comprises a conserved, modular core sequence interspersed by highly variable cargo genes [57]. The conserved core sequence is made up of three distinct modules encoding replication, transfer and integration genes [6]. The transfer region in well-characterised ICEs is homologous to the LGI-T4SS cluster and encodes a GI-T4SS that represents the conjugative component of this mobile element. To investigate if the replication and integration modules were also found associated with the LGIs, we compared the LGIs to three well-characterised SPI-7 family ICEs, ICE*Hin*1056 (*H. influenzae*), PAPI-1 (*P. aeruginosa*) and ICE*Sb*1 (*S. bongori*) [6], [11], [18] which were found to encode similar GI-T4SSs (Figure 2). The integrases of the other characterised ICEs in this family share limited but significant sequence similarity to one another (25–30% amino acid identity) even though they recognise and integrate into different tRNA genes (ICE*Sb*1: tRNA^Phe^, PAPI-1: tRNA^Lys^, ICE*Hin*1056: tRNA^Leu^) [11], [63], [64]. Our analyses clearly showed that the replication and integration modules found in these well-characterised ICEs do not have homologues in the LGIs (Table 2).

Although the genes on the LGIs lack any similarity to the relaxase (*traI*) or integrase (*int*) which comprise the integration module in the characterised ICEs [11], [65], we were able to identify unrelated putative integrase genes based on the presence of conserved integrase domains in CDSs adjacent to the insertion site of each of the LGIs, but no substitute for a relaxase could be identified, apart from a 174 bp fragment, present only in LppGI-2, matching the first 50 amino acids of the relaxase TraA found in the Lvh island of *L. pneumophila* Philadelphia 1.

An integrase gene was identified close to the putative insertion sites of each LGI-2 (Figures S2, Table S6) and it is possible that these integrase genes form at least part of an integration module. Supporting this hypothesis, this integrase gene in LpcGI-2 (LPC_1833) was recently shown to be required for the excision of LpcGI-2 in Corby [28]. Moreover, this integrase is also associated with six to seven other predicted genes conserved only in LGI-2s (Figure S2). This cluster includes genes that putatively encode two acetyl-transferases, a transcriptional regulator and a proline transport protein (Table S6). Among the LGI-1 islands, another unrelated putative integrase gene is also conserved adjacent to the *attR* (first repeat, see Figure S1). This integrase is also conserved alongside seven genes, which putatively code for two transcription regulators, a peptide deformylase and a proline transport protein (Table S6). This LGI-1 specific region is not found in any of the other LGIs. Finally, a tandem pair of putative integrases was also found between 1 to 17 Kb downstream from 13 of the 14 LGI clusters (Figures S1 and S2). The deletion of one of these integrases in strain Corby was shown to significantly increase the presence of the episomal form of the island [28]. However, the reason for this observation is as yet unknown.

Relaxase genes, also known as mobilisation genes *(mob)*, are believed to be essential for the processing of the DNA fragment prior to and after conjugation. Relaxases nick the excised, circular, double-stranded DNA fragment at the origin of transfer (*oriT*) and then bind to it. The entire relaxase-DNA complex is then coupled to the T4SS by the coupling protein (VirD4/TraD) and is subsequently transferred into the recipient cell. Once in the recipient cell, the newly acquired DNA fragment is ligated by the relaxase [66]. Given the apparent absence of both an *oriT* and conserved relaxase associated with the LGIs, it is perhaps surprising that conjugation of the genomic island encoding LpcGI-2 was recently demonstrated in strain Corby [28]. However, it is possible that there are yet unidentified interactions between the LGI and relaxases and replication genes encoded elsewhere in the genome, as it has previously been shown that different T4SS encoded on the same genome are able to ‘share’ missing subunits [1], [26], [27]. Indeed, in *Vibrio*, an unrelated family of ICEs, the SXT/R391 family, was shown to mobilise and mediate conjugation of other genomic islands located elsewhere in the genome. Utilising the relaxase and conjugation machinery encoded by an SXT element, *Vibrio* genomic islands lacking their own native relaxase and conjugation system could excise and transfer conjugatively, but required a conserved relaxase recognition site (*oriT*) to be present [67]. However, these mobilised *Vibrio* genomic islands, unlike the LGIs, did not encode their own T4SS.

The replication module in the SPI-7 ICE family is made up of at least seven genes with some variation in gene content between different elements. Six of these highly conserved genes are similar to plasmid replication and maintenance genes (*parA*, *dnaB*, *parB*, *ssb*, *topB* and *radC*) while others are conserved hypothetical genes [7]. In ICE*Sb*1, the deletion of eight genes found within the replication module resulted in a significant reduction of conjugation frequencies (Table 2) [11]. In the LGIs, the only genes with any similarity to those found in the replication module were the genes encoding topoisomerase (*topB*) and the single stranded binding protein (*ssb*). The *ssb* genes were only found on LGI-1 in the *L. pneumophila* strains and LdrGI-c in *L. drancourtii*, while *topB* was only found on LdrGI-c and LdrGI-d. These two genes were not essential for the conjugation of this element in ICE*Sb*1 [11] and their lack of conservation in all LGI clusters makes *ssb* and *topB* unlikely to be part of the missing replication module.

Manual annotation of the LGIs in the complete genomes identified a number of genes involved in DNA replication that were scattered across the region (Table S6). Two genes located between the tRNA gene and the LGI-1 T4SS cluster (lpp1039 and lpp1040, lpl1004 and lpl1005, LPW_10611 and LPW_10621, lpa_01469 and lpa_01470), putatively encode UmuD and UmuC which together form the DNA polymerase V holoenzyme that is implicated in DNA replication during an SOS response as result of DNA damage [68]. The SOS response is known to induce the excision of other unrelated ICEs [69], [70]. During this process DNA polymerase V also interacts with the single-stranded binding protein, which is putatively encoded by the *ssb* gene located 3.5 Kb downstream [71]. These two genes, *umuD* and *umuC*, however, were only present in four of the six LGI-1 regions. We also identified several putative helicases (lpl1068, lpl1073, lpa_01670, lpa_01676, LPC_2195, lpg1077, LDG_0893) and excisionases (lpa_01673, LPC_2192, lpg1081) although again, these are not consistently conserved across all LGIs.

Despite the apparent absence of a universally conserved replication module, the ability of LpcGI-2 to excise from the genome and transfer conjugatively suggests that this module is not required in *Legionella*, especially as no replication-associated genes were identified in LpcGI-2 (Table S6). The specific role that the replication region plays in GI-T4SS associated ICEs is still largely unknown, although multiple genes encoded in the replication module were shown to be essential for conjugative transfer of the element in ICE*Sb*1 and ICE*Hin*1056 [11], [72]. In addition, unrelated ICEs such as ICE*Bs*1 in *Bacillus subtilis* have been shown to use either self-encoded or bacterial host helicases for unwinding of the excised extrachromosomal DNA prior to both autonomous replication and conjugation [73], [74].

### Several putative cargo genes are encoded on the LGIs

BLAST analyses of the CDSs within the LGIs against the NCBI non-redundant sequence database indicated diverse origins of the genes encoded in the hypervariable regions flanking the LGI-T4SSs. Some CDSs had their closest match in the Uniprot database to genes from other phyla of bacteria including cyanobacteria and firmicutes. Other CDSs were related to those found in alpha and beta-proteobacteria but were not found in other gamma-proteobacteria. This observation is typical of horizontally acquired regions which are modular and mosaic as they are often derived from different mobile genetic elements [75]. Putative integrases and transposases are scattered throughout the LGIs (Figures S1 and S2) supporting the hypothesis that the genomic loci flanking the LGI-T4SS are the result of multiple insertion and deletion events, as is commonly found in ICEs [12]. Variable cargo regions, in some cases of considerable length, are usually found interspersed between the core transfer, replication and integration modules in ICEs, and they often encode factors that increase the fitness or virulence of the host bacterium e.g. the Vi-encoding operon in SPI-7 [11]. Therefore it is likely that the hypervariable regions in the LGIs represent the ICE cargo regions. Indeed there are a number of antibiotic resistance and virulence genes encoded within these genomic islands including five Dot/Icm effectors (LPC_2130, LPC_2128, lpp2417, lpp2418 and lpw_21901), as well as several genes encoding beta lactamase and aminoglycoside resistance (Table S6). Of note are a number of putative P-type ATPases and resistance/nodulation/division (RND) efflux systems, with up to five versions of each per LGI (Figures S1 and S2, Table S6). This was first identified as an ‘efflux island’ in the Philadelphia genome [31]. A great deal of divergence is seen between the different versions of the ATPase genes and RND operons (Figures S1 and S2), which suggests there may be distinct functions or target substrates for each of these transport systems. One of the efflux systems encoded on LpgGI-1 (lpg1024) has previously been shown to confer copper resistance, although substrates have not been identified for the others, and due to the absence of a signal sequence, it is difficult to determine the exact cation or substrate that could be translocated by the others [76]. RND efflux systems in *Escherichia coli*, *Pseudomonas aeruginosa* and *Vibrio cholerae* have been shown to be involved in antibiotic resistance and pathogenicity [77]–[79], and strong simultaneous up-regulation of these efflux genes during *L. pneumophila* infection has previously been reported [80]. Although a study found that the RND systems were not essential for the survival of *L. pneumophila* Philadelphia in amoeba and human macrophages [76], only four of the RND operons found near LpgGI-1 were knocked out, leaving intact additional RND-type systems encoded elsewhere in the Philadelphia genome. Therefore the role of these putative efflux systems in *L. pneumophila* remains to be determined, but it is possible that they confer a fitness advantage to *Legionella* during infection.

## Conclusions

Our results show that the LGI-T4SS is well conserved throughout the *Legionella* genus and belongs to a distinct and divergent lineage within the GI-T4SS clade, exhibiting features that are unique to this lineage such as the association with *Legionella*-specific regulatory genes. The presence of more than one LGI-T4SS in a single genome, each under purifying selection, indicates that these clusters play an active and important role in this organism. Therefore, despite the association with highly variable LGI regions and the significant level of divergence from known ICE, the LGI-T4SS clusters are likely to be functional and involved in horizontal gene transfer via conjugation. The LGI-T4SSs are each encoded on different genomic islands, which were likely acquired horizontally and integrated into the adjacent tRNA genes. The structure of the LGIs show significant similarity to characterised ICEs, including direct flanking repeats, a conjugation (GI-T4SS) module, putative integration module and highly variable cargo regions. Investigation of additional *Legionella* genomes that have been published recently, those of *Legionella pneumophila* strains LPE509, HL06041035, Lorraine, 570-CO-H, and Thunder Bay [22], [81]–[83], concur with these results, as one or two LGI-T4SSs were identified per genome, each encoded on an ICE-like LGI (Table S7).

Our findings correspond with the fact that all GI-T4SSs characterised to date are associated with ICEs. However, no replication module or conserved replication genes were identified on the LGIs, and there was no significant sequence similarity with the integration modules usually closely associated with other GI-T4SS, supporting the atypical arrangement of the LGIs.

Despite the absence of these typical ICE modules, LpcGI-2 was recently experimentally demonstrated to be a functional ICE [28]. The similarity of the putative integration and conjugation modules between the different LGIs, together with the flanking repeats that represent potential *attL* and *attR* sites, indicate that the other LGIs may also be functional ICEs. Concordant with this, excision of LpgGI-1 was recently demonstrated, with increased copper resistance on excision [84]. The absence of typical ICE modules involved in the processing of DNA prior to conjugation suggests that there could be an atypical mechanism of preprocessing involved with the mobilisation and conjugation of the LGIs.

The LGI-T4SSs are likely to contribute to horizontal transmission in *Legionella*, including transfer of the many cargo genes carried on the LGIs, which include several antibiotic resistance and virulence genes, and as such are key players in the evolution of *Legionella*. Therefore, future investigations of virulence and the roles of the many T4SSs encoded in this opportunistic pathogen should take into account this unusual member of the GI-T4SS family.

## Supporting Information

Figure S1 **Comparison of LGI-1 genomic islands.** Pairwise comparisons of the genomic regions encoding the LGI-1 clusters from the genomes of *L. pneumophila* strains Philadelphia, Corby, Alcoy, 130b, Lens and Paris (from top to bottom). The grey bars indicate BLASTn hits between two adjacent sequences, shaded according to the percentage nucleotide sequence identity, as shown in the key. CDSs are represented as arrows coloured according to putative functional category as defined in the key. The scale bar represents 10 Kb.
(PDF)

Figure S2 **Comparison of the LGI-2 genomic islands.** Pairwise comparisons of the second LGIs from the genomes of *L. pneumophila* strains Corby (LpcGI-2), Paris (LppGI-2), and 130b (LpwGI-2), and *L. drancourtii* (LdrGI-a). The grey bars indicate BLASTn hits between two adjacent sequences, shaded according to the percentage nucleotide sequence identity, as shown in the key. CDSs are represented as arrows coloured according to putative functional category as defined in the key. The scale bar represents 10 Kb.
(PDF)

Figure S3 **Graphical representation of recombination events in LGI-T4SSs.** Recombination events, detected using RDP, in the concatenated sequence of 15 conserved *lgi* genes. The position of each gene in the alignment is displayed above and below the figure with letters corresponding to *lgi* gene names. The total alignment length is 12 Kb.
(PDF)

Table S1 **Annotation of LGI-T4SS genes.**
(XLS)

Table S2 **Average nucleotide identity (ANI) between LGI-T4SS clusters (%).**
(DOC)

Table S3 **Whole Genome ANI (%) between the nine** ***Legionella*** **genomes analysed in this study.**
(DOC)

Table S4 **Recombination events detected by the RDP package supported by at least two methods and larger than 500 bp.**
(DOC)

Table S5 **Comparisons of estimated dN/dS values for different sets of LGI-T4SS clusters.**
(XLS)

Table S6 **Annotation of genes encoded within ten LGIs (as shown in Figures S1 and S2).**
(XLS)

Table S7 **Distribution of GI-T4SSs and associated LGIs in new** ***Legionella*** **genomes published since commencement of this study.**
(XLS)
