# Supplementary figures and images for: A Distinct and Divergent Lineage of Genomic Island-Associated Type IV Secretion Systems in Legionella
Source: PLoS One. 2013 Dec 16;8(12):e82221. doi: 10.1371/journal.pone.0082221 (PMC3864950; doi:10.1371/journal.pone.0082221)

**Figure S1** Comparison of LGI-1 genomic islands.

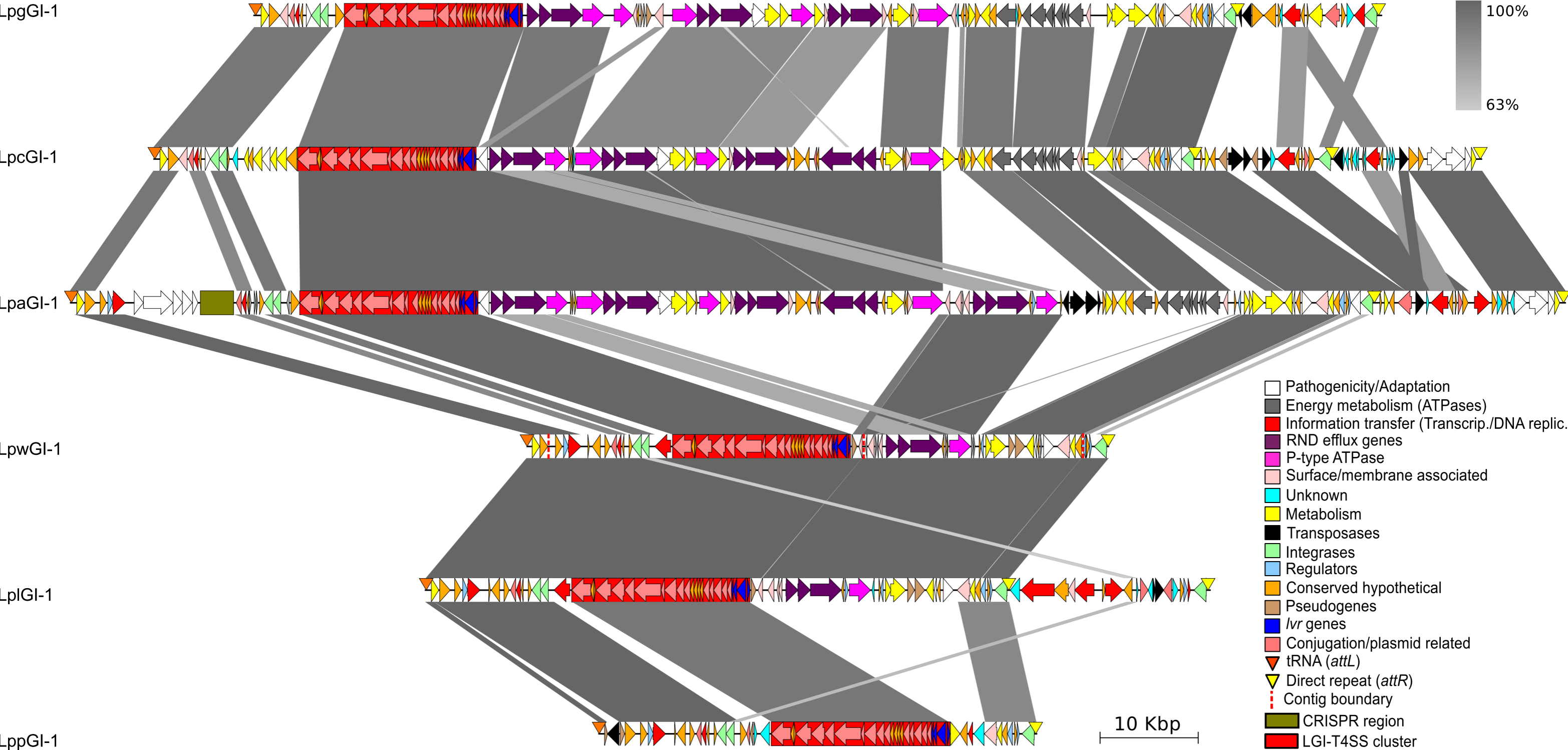

Supplement: Figure S1 — Comparison of LGI-1 genomic islands. Pairwise comparisons of the genomic regions encoding the LGI-1 clusters from the genomes of L. pneumophila strains Philadelphia, Corby, Alcoy, 130b, Lens and Paris (from top to bottom). The grey bars indicate BLASTn hits between two adjacent sequences, shaded according to the percentage nucleotide sequence identity, as shown in the key. CDSs are represented as arrows coloured according to putative functional category as defined in the key. The scale bar represents 10 Kb. (PDF) [file pone.0082221.s001.pdf]

**Figure S2** Comparison of the LGI-2 genomic islands.

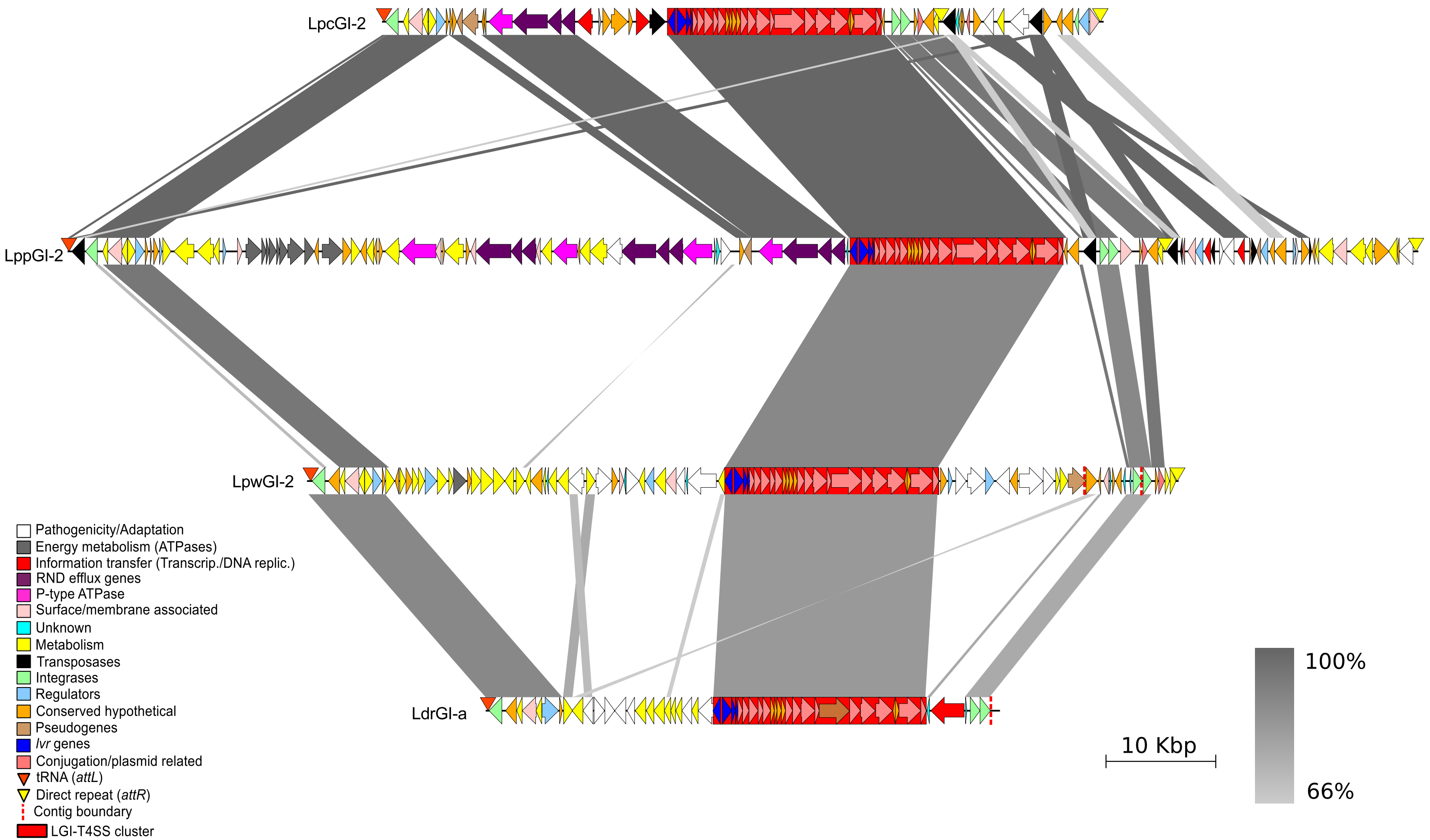

Supplement: Figure S2 — Comparison of the LGI-2 genomic islands. Pairwise comparisons of the second LGIs from the genomes of L. pneumophila strains Corby (LpcGI-2), Paris (LppGI-2), and 130b (LpwGI-2), and L. drancourtii (LdrGI-a). The grey bars indicate BLASTn hits between two adjacent sequences, shaded according to the percentage nucleotide sequence identity, as shown in the key. CDSs are represented as arrows coloured according to putative functional category as defined in the key. The scale bar represents 10 Kb. (PDF) [file pone.0082221.s002.pdf]

**Figure S3** Graphical representation of recombination events in LGI-T4SSs.

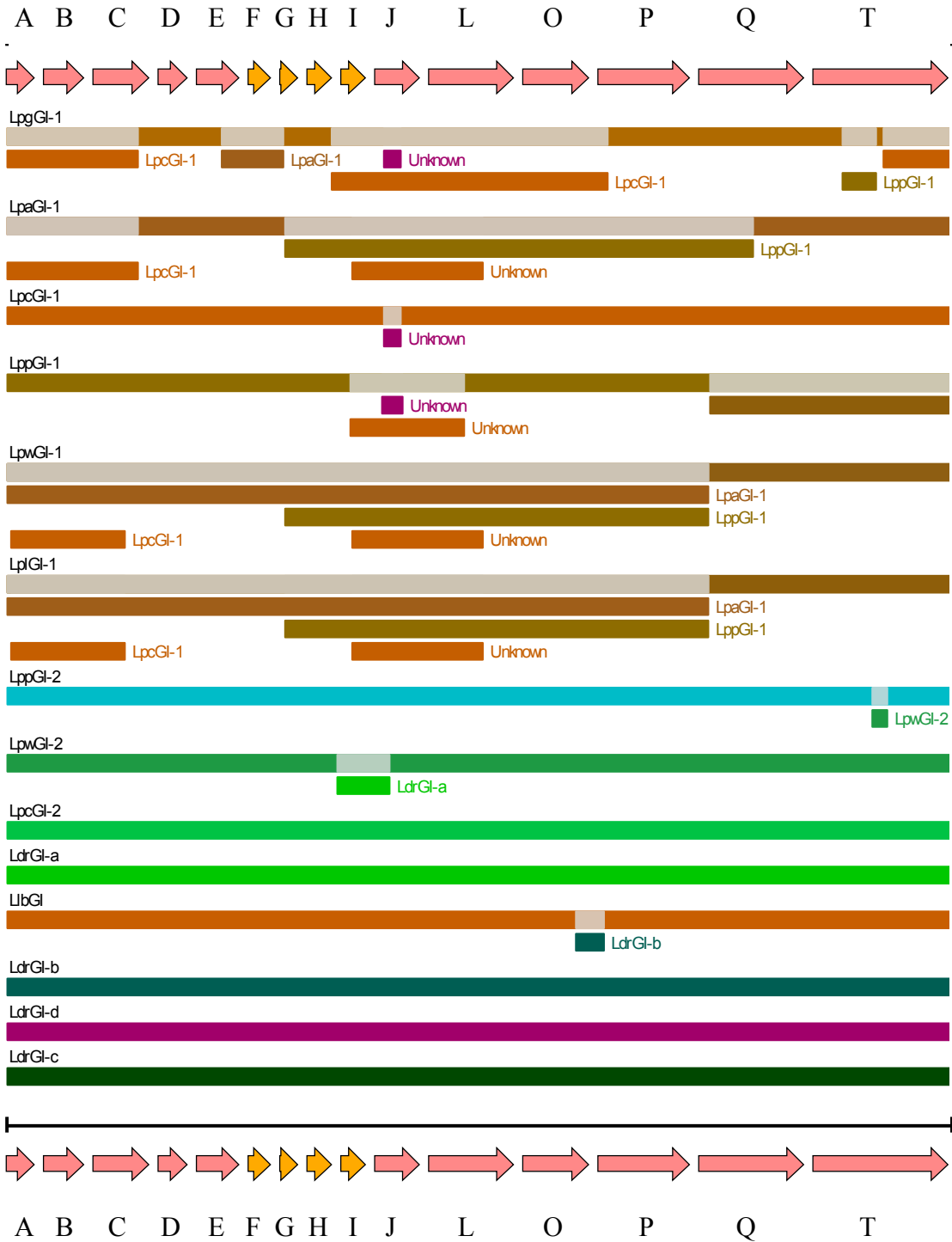

Supplement: Figure S3 — Graphical representation of recombination events in LGI-T4SSs. Recombination events, detected using RDP, in the concatenated sequence of 15 conserved lgi genes. The position of each gene in the alignment is displayed above and below the figure with letters corresponding to lgi gene names. The total alignment length is 12 Kb. (PDF) [file pone.0082221.s003.pdf]
