# Supplementary material for: A Distinct and Divergent Lineage of Genomic Island-Associated Type IV Secretion Systems in Legionella
Source: PLoS One. 2013 Dec 16;8(12):e82221. doi: 10.1371/journal.pone.0082221 (PMC3864950; doi:10.1371/journal.pone.0082221)
Supplement: Table S2 — Average nucleotide identity (ANI) between LGI-T4SS clusters (%). (DOC) [file pone.0082221.s005.doc]

| **Table S2. Average nucleotide identity (ANI) between LGI-T4SS clusters (%).** | | | | | | | | | | | | |  |  |
| --- | --- | --- | --- | --- | --- | --- | --- | --- | --- | --- | --- | --- | --- | --- |
|  |  |  |  |  |  |  |  |  |  |  |  |  |  |  |
|  | LpaGI-1 | LpcGI-1 | LpgGI-1 | LplGI-1 | LppGI-1 | LpwGI-1 | LpcGI-2 | LppGI-2 | LpwGI-2 | LdrGI-a | LdrGI-b | LlbGI | LdrGI-c | LdrGI-d |
| LpaGI-1 | --- | 93.1 | 93.5 | 96.8 | 92.1 | 96.8 | 69.3 | 68.3 | 69.0 | 67.6 | 68.3 | 68.1 | 65.1 | 64.1 |
| LpcGI-1 | 93.1 | --- | 93.8 | 93.1 | 89.4 | 93.1 | 69.3 | 68.3 | 68.9 | 67.9 | 68.7 | 68.6 | 64.7 | 63.3 |
| LpgGI-1 | 93.5 | 93.8 | --- | 92.8 | 89.5 | 92.8 | 67.8 | 67.8 | 69.2 | 67.2 | 68.7 | 67.9 | 64.3 | 63.9 |
| LplGI-1 | 96.7 | 93.1 | 92.8 | --- | 94.1 | 100.0 | 68.6 | 68.5 | 69.1 | 67.9 | 68.4 | 68.0 | 65.2 | 63.0 |
| LppGI-1 | 92.0 | 89.5 | 89.5 | 94.1 | --- | 94.1 | 69.7 | 68.5 | 69.6 | 68.0 | 69.0 | 68.5 | 64.1 | 63.1 |
| LpwGI-1 | 96.8 | 93.1 | 92.8 | 100.0 | 94.0 | --- | 68.5 | 68.4 | 69.0 | 67.9 | 68.3 | 68.0 | 65.2 | 62.9 |
| LpcGI-2 | 68.9 | 68.9 | 68.8 | 68.9 | 68.9 | 68.9 | --- | 96.5 | 85.2 | 79.5 | 72.0 | 74.4 | 65.2 | 63.4 |
| LppGI-2 | 67.6 | 68.7 | 68.0 | 67.8 | 67.8 | 67.8 | 96.5 | --- | 85.3 | 79.5 | 71.9 | 74.6 | 64.8 | 63.3 |
| LpwGI-2 | 68.5 | 68.6 | 68.3 | 68.7 | 68.1 | 68.7 | 85.3 | 85.3 | --- | 80.5 | 72.3 | 75.7 | 66.0 | 64.6 |
| LdrGI-a | 68.1 | 68.5 | 68.3 | 68.6 | 68.5 | 68.6 | 79.6 | 79.6 | 80.5 | --- | 72.5 | 75.6 | 65.8 | 66.2 |
| LdrGI-b | 66.4 | 66.4 | 66.0 | 66.5 | 66.7 | 66.5 | 71.9 | 71.9 | 72.2 | 72.2 | --- | 74.1 | 65.4 | 63.9 |
| LlbGI | 67.9 | 67.9 | 67.8 | 67.9 | 67.2 | 67.9 | 74.8 | 73.9 | 74.9 | 75.5 | 73.9 | --- | 65.4 | 63.6 |
| LdrGI-c | 64.7 | 65.6 | 65.7 | 64.9 | 65.6 | 64.9 | 66.2 | 65.9 | 66.0 | 66.3 | 67.0 | 66.9 | --- | 69.2 |
| LdrGI-d | 64.1 | 64.4 | 64.0 | 64.6 | 64.7 | 64.6 | 64.8 | 64.7 | 64.9 | 65.7 | 65.7 | 64.7 | 69.4 | --- |
