# Supplementary material for: A Distinct and Divergent Lineage of Genomic Island-Associated Type IV Secretion Systems in Legionella
Source: PLoS One. 2013 Dec 16;8(12):e82221. doi: 10.1371/journal.pone.0082221 (PMC3864950; doi:10.1371/journal.pone.0082221)
Supplement: Table S3 — Whole Genome ANI (%) between the nine Legionella genomes analysed in this study. (DOC) [file pone.0082221.s006.doc]

**Table S3. Whole Genome ANI (%) between the nine *Legionella* genomes analysed in this study.**

|  | Lpa | Lpc | Lpg | Lpp | Lpl | Lpw | Llo | Llb | Ldr |
| --- | --- | --- | --- | --- | --- | --- | --- | --- | --- |
| *Legionella pneumophila* Alcoy (Lpa) | --- | 99.4 | 96.7 | 97.3 | 96.3 | 96.4 | 71.7 | 71.5 | 72.1 |
| *L. pneumophila* Corby (Lpc) | 99.3 | --- | 96.5 | 97.3 | 96.0 | 96.2 | 71.7 | 71.6 | 72.4 |
| *L. pneumophila* Phila 1(Lpg) | 96.7 | 96.7 | --- | 96.9 | 96.6 | 96.9 | 71.8 | 71.7 | 72.0 |
| *L. pneumophila* Paris (Lpp) | 97.2 | 97.3 | 96.8 | --- | 96.3 | 96.5 | 71.6 | 71.6 | 72.1 |
| *L. pneumophila* Lens (Lpl) | 96.4 | 96.3 | 96.7 | 96.5 | --- | 98.4 | 71.9 | 71.9 | 72.0 |
| *L. pneumophila* 130b (Lpw) | 96.4 | 96.4 | 96.6 | 96.4 | 98.1 | --- | 71.8 | 71.6 | 72.2 |
| *L. longbeachae* NSW-150 (Llo) | 71.8 | 71.7 | 71.7 | 71.6 | 72.0 | 71.9 | --- | 99.8 | 74.8 |
| *L. longbeachae* D-4968 (Llb) | 71.6 | 71.6 | 71.7 | 71.5 | 71.9 | 71.6 | 99.8 | --- | 74.6 |
| *L. drancourtii* LLAP12 (Ldr) | 71.8 | 72.0 | 71.5 | 71.8 | 71.7 | 72.1 | 74.4 | 74.2 | --- |
