# Supplementary material for: A Distinct and Divergent Lineage of Genomic Island-Associated Type IV Secretion Systems in Legionella
Source: PLoS One. 2013 Dec 16;8(12):e82221. doi: 10.1371/journal.pone.0082221 (PMC3864950; doi:10.1371/journal.pone.0082221)
Supplement: Table S4 — Recombination events detected by the RDP package supported by at least two methods and larger than 500 bp. (DOC) [file pone.0082221.s007.doc]

**Table S4. Recombination events detected by the RDP package supported by at least two methods and larger than 500bp.**

| **Event Number** | **Clusters affected by Recombination** | **Putative size of recombinant fragment (Kb)** | **P-value (Corrected for multiple testing)** | **Methods identifying the event** |
| --- | --- | --- | --- | --- |
| 1 | LpaGI-1, LplGI-1, LpwGI-1 | 5.7 | 7.00E-124 | RDP, GENECONV, BootScan, MaxChi, Chimaera, Siscan |
| 2 | LpaGI-1, LpwGI-1, LplGI-1, LppGI-1 | 4.3 | 1.00E-68 | RDP, GENECONV, BootScan, MaxChi, Chimaera, Siscan |
| 3 | LpgGI-1, LpcGI-1 | 2.8 | 1.00E-52 | RDP, GENECONV, BootScan, MaxChi, Chimaera, Siscan |
| 4 | LpgGI-1, LppGI-1, LpcGI-1, LpaGI-1, LpwGI-1, LplGI-1 | 1.2 | 4.00E-09 | RDP, GENECONV, BootScan, MaxChi, Chimaera, Siscan |
| 5 | LpaGI-1, LppGI-1, | 1 | 7.00E-11 | RDP, GENECONV, BootScan, MaxChi, Chimaera |
| 6 | LppGI-1, LpwGI-1, LpaGI-1 | 1 | 1.00E-09 | GENECONV, Chimaera, Siscan |
| 7 | LppGI-1, LplGI-1, LpcGI-1 | 5.3 | 4.00E-14 | RDP, GENECONV, BootScan, Chimaera, Siscan |
